# Reply to: Beyond AUC: Advancing clinical interpretability of depression–MRI–machine learning analyses in Alzheimer's disease

**DOI:** 10.1002/alz.71439

**Published:** 2026-05-03

**Authors:** Chao Tang, Jiaxin Yang, Xiaoyang Lei, Dian He

**Affiliations:** ^1^ Department of Neurology Affiliated Hospital of Guizhou Medical University Guiyang Guizhou Province China

**Keywords:** Alzheimer's disease, clinical utility, depression, machine learning, magnetic resonance imaging

Dear Editor,

We appreciate the thoughtful and constructive comments from Dr. Deng and Dr. Xie regarding our study, “Association between brain volume and depression in Alzheimer's disease: Neuroimaging insights.”^1^ Their proposed “Beyond AUC” framework provides a critical roadmap for transitioning from neuroimaging‐based discovery to clinically implementable decision support tools.^2^ As the authors of the original study, we welcome this opportunity to discuss how our findings provide a foundation for the refinements they suggest.

## 1 REFINING DEPRESSION PHENOTYPING IN DEMENTIA

Deng and Xie correctly point out that a single Geriatric Depression Scale (GDS) cut‐point may not fully capture the complex overlap between depression, apathy, and sleep disturbances in Alzheimer's disease (AD). In our analysis of 2722 participants, we utilized the GDS due to its consistent availability across the multi‐site National Alzheimer's Coordinating Center (NACC) database. Although this ensured a robust sample size for establishing the mediation effect of hippocampal atrophy (indirect effect = −0.107, *p* < 0.001), we agree that “triangulation” is essential. Future work within the NACC framework should indeed cross‐validate GDS‐defined symptoms against clinician diagnoses or the Cornell Scale for Depression in Dementia (CSDD), which remains the gold standard for neurodegenerative populations.

## 2 ADDRESSING SPECTRUM EFFECTS AND CLINICAL GENERALIZABILITY

Our study utilized a case–control design (Cognitively Normal [CN] with Mini‐Mental State Examination [MMSE] Transparent Reporting of a multivariable prediction model for Individual Prognosis Or Diagnosis for Artificial Intelligence score ≥27 vs AD with MMSE ≤24) to isolate clear neuroanatomic signals. However, clinical translation requires performance validation in “boundary states” such as mild cognitive impairment (MCI). We agree that evaluating whether our machine learning (ML) models (area under the curve [AUC] 0.871 for AD classification; *R^2^* = 0.625 for depression prediction) maintain utility in these populations is a priority. Spectrum effects are decisive; clarifying how brain–depression associations evolve from MCI to early AD will reveal whether these markers are early warning signs or late‐stage manifestations of disease.

## 3 CAUSAL ESTIMANDS AND THE “MMSE PARADOX”

A particularly salient statistical point was raised regarding our adjustment for MMSE scores. We included MMSE to control for global cognitive severity while examining depression‐specific brain changes. However, as noted, if cognitive decline lies on the causal path between neurodegeneration and mood, adjusting for MMSE may estimate a “direct effect” rather than a “total effect,” potentially introducing “collider bias.” We endorse the use of directed acyclic graphs (DAGs) in future research to explicitly define the intended estimand and ensure that covariate selection is biologically and statistically motivated.

## 4 FROM DISCRIMINATION TO CLINICAL UTILITY

Deng and Xie advocate for a paradigm shift from discrimination (AUC) to clinical net benefit. We agree that reporting metrics such as calibration (the accuracy of predicted probabilities) and Brier scores is essential for clinical trust. Furthermore, integrating decision curve analysis (DCA) would allow us to quantify whether our ML models provide added value over default clinical strategies at specific intervention thresholds. Adherence to updated reporting standards, such as Transparent Reporting of a multivariable prediction model for Individual Prognosis Or Diagnosis for Artificial Intelligence (TRIPOD+AI) and Prediction model Risk Of Bias ASsessment Tool for Artificial Intelligence (PROBAST+AI), will be instrumental in ensuring the transparency and rigor of these high‐dimensional models.

In conclusion, our original study identified hippocampal atrophy as a core neurobiological link between depression and AD. We share the vision of Deng and Xie for the next stage of this research. By refining our phenotyping and embracing decision‐analytic evaluations, we can bridge the gap between “statistical significance” and “clinical excellence.”

## CONFLICT OF INTEREST STATEMENT

The authors declare no conflicts of interest. Author disclosures are available in the Supporting Information.

## FUNDING INFORMATION

This work was supported by the STI2023‐Major Projects (2021ZD0201801); the Key and Dominant Discipline Construction Project of the Health Commission of Guizhou Province in 2023, China; the Guizhou Provincial Science and Technology Support Program Project (General item: 2025‐117); and the Guizhou Provincial Clinical Medical Research Center Construction Project—Neurological Disease Research (No: LCZX[2025]003).

## Supporting information



Supporting Information
